# Emergency cesarean section of a patient with refractory status epilepticus: A case report of challenges during anesthesia

**DOI:** 10.1097/MD.0000000000037988

**Published:** 2024-05-03

**Authors:** Jin Soo Park, Jin Hun Chung, Nan Seol Kim, Ho Soon Jung, Yong Han Seo, Hyung Youn Gong, Jae Yong Ji, Yu Jun Park, Jun Yong Jung, Hea Rim Chun

**Affiliations:** a Department of Anesthesiology and Pain Medicine, Affiliated Soonchunhyang University Hospital Cheonan, Cheonan-si, Chungcheongnam-do, Republic of Korea.

**Keywords:** anesthetic management, case report, epilepsy, preeclampsia, status epilepticus

## Abstract

**Introduction:**

Maternal epilepsy is a critical condition that can significantly affect mothers and fetuses. Notably, the admission of a laboring mother with uncontrolled refractory status epilepticus (RSE) to the operating room presents a challenging scenario for anesthesiologists.

**The main symptoms of the patient and the important clinical findings:**

A 30-year-old primigravida was transferred to the operating room for an emergency cesarean section. Cesarean section was performed after a provisional diagnosis of preeclampsia was made.

**The main diagnoses, therapeutic interventions, and outcomes:**

Cesarean section was performed under general anesthesia. During the postoperative period, the patient exhibited no seizure activity in the brain; however, she experienced mild cognitive dysfunction for up to 6 months postdelivery. The neonate were discharged without any complications.

**Conclusion:**

Inducing anesthesia in pregnant women with ongoing seizure activity are challenging; however, anesthesiologists provide judgment based on the balance between the safety of the mother and fetus and the balance between patient monitoring and the progression of anesthesia. This challenge can be addressed through multidisciplinary collaboration.

## 1. Introduction

This study presents a case of a 30-year-old pregnant patient with refractory status epilepticus (RSE) who showed uncontrolled seizures in the operation theater; this scenario is rarely encountered. This report presents the complexities and critical decision-making involved in anesthetic management of high-risk cases. We emphasize the need for increased awareness and readiness in managing similar perinatal situations by describing this patient’s treatment and the interdisciplinary strategy used. Our study significantly contributes to the growing body of knowledge in this field and enhances the care standards of mothers and neonates under such conditions.

## 2. Case presentation

A 30-year-old primigravida at 30 weeks gestation who presented with impaired consciousness and ongoing generalized tonic–clonic seizures were admitted. Based on the assessment of the last known normal time and the first abnormal time, seizures were estimated to have persisted for up to 4 hours. The patient had no history of seizures; however, she had been managing gestational diabetes using oral medications. On admission, her blood glucose level was 40 mg/dL, which could have been due to impaired consciousness or a prolonged seizure episode. Hypoglycemia was corrected by administering 50% dextrose. Additionally, urine analysis revealed proteinuria. Despite aggressive treatment with intravenous lorazepam, levetiracetam, and magnesium sulfate, the seizures persisted. To prevent the complications of fetal stress, obstetricians made an urgent decision to perform cesarean delivery. Preoperative CT scan of the brain was conducted to confirm the absence of neurosurgical emergencies such as hemorrhage, and the patient was transferred to the operating theater immediately.

The generalized tonic movements persisted in the operating room. General anesthesia was induced by the anesthesiology team using propofol at a dose of 1.5 mg/kg and a maintenance rate of 4 mg/kg/h. The tonic movements subsided following propofol administration, after which 50 mg of intravenous rocuronium was administered, enabling successful endotracheal intubation. During the procedure, controlled ventilation was monitored to maintain end-tidal CO_2_ (ETCO_2_) levels at 40 mm Hg.

Invasive arterial blood pressure, pulse oximetry, electrocardiography, peripheral nerve stimulation, and esophageal temperature were monitored throughout the surgery. In addition, processed electroencephalogram (pEEG) was monitored using the Sedline monitor. Initially, the tonic movements of the patient compromised the functioning of the Sedline monitor; however, proper functioning of the device was achieved after neuromuscular blockade with rocuronium. Notably, patient state index levels were maintained at 40 to 50 postneuromuscular blockade.

Following delivery, the newborn exhibited cyanosis and showed a 1-minute Apgar score of 5; immediate tracheal intubation was performed. Intubation improved the Apgar score to 7 at the 5-minute assessment. After completion of the cesarean section procedure, the patient was transferred to the ICU for ongoing mechanical ventilation. A neurologist initiated sedative tapering and ventilator weaning. The patient was successfully weaned off mechanical ventilation on the first postoperative day, and continuous EEG monitoring revealed no abnormalities.

The patient regained consciousness, gradually reorientated, and responded to simple commands during the early recovery period. The neurologist conducted tests, including Brain MRI and autoimmune assays, to determine the cause of RSE, and all tests revealed no significant abnormalities. After a month of hospitalization, she was discharged; however, her cognitive function was not fully restored. The patient responded to simple commands and was transferred to a rehabilitation facility for further care.

Follow-up assessments at 6 months after discharge revealed a mini-mental state examination score of 21 and a geriatric depression scale score of 4, indicating mild cognitive impairment. The newborn showed normal development. This case report was prepared per the CARE guidelines, ensuring comprehensive and transparent reporting of all relevant aspects of the case.

## 3. Discussion

This case study focused on anesthetic management in pregnancy-related SE, a rare disease that affects 0.6% of pregnant patients with epilepsy.^[1]^

Although the patient had no history of seizures, status epilepticus during pregnancy is not uncommon. Pregnancy-associated SE primarily occurs in patients without a history of epilepsy^[2]^; typical causes include cortical venous thrombosis, eclampsia, and posterior reversible encephalopathy syndrome.^[3]^ Here, the patient was diagnosed with eclampsia based on imaging and laboratory tests. The patient exhibited persistent cognitive dysfunction, likely due to delayed detection and prolonged SE duration.

When managing RSE during a cesarean section, the anesthetic strategy should be carefully considered. Benzodiazepines are the first choice for SE management in pregnancy, except in cases of eclampsia^[4]^; patients are likely to experience benzodiazepine-induced sedation. However, in cases of eclampsia, magnesium sulfate is administered as the initial treatment because it effectively reduces maternal mortality and seizure recurrence compared with diazepam.^[5]^ The treatment approach and choice of anesthetic strategy significantly depend on the underlying cause of SE.

Notably, there is a widespread shift away from general anesthesia in cesarean sections; however, when employed, agents such as propofol, barbiturates, and midazolam are preferred. These are third-line antiepileptic drugs and standard anesthetics; therefore, these agents offer a dual benefit. Anesthesiologists must carefully navigate between antiepileptic and anesthetic doses.

Propofol is the most commonly used agent for total intravenous anesthesia, which allows for convenient dosage determination. Table 1 shows the typical doses of propofol used under general anesthesia with the Massachusetts general hospital neurology status epilepticus treatment protocol.^[6]^ As there is considerable overlap in the therapeutic dose range, anesthesiologists can concurrently manage anesthesia induction and interruption of SE seizures without significantly deviating from the usual dosages of propofol.

**Table 1:** Recommended propofol doses for general anesthesia induction and the Massachusetts general hospital neurology status epilepticus treatment protocol.

Propofol dose for general anesthesia (for adults aged < 55 yr and classified as ASA-PS I OR II) Induction of general anesthesia: 2 to 2.5 mg/kg IV Maintenance infusion: 6 to 12 mg/kg/h
Propofol dose recommended by the MGH status epilepticus treatment protocol (6) Loading dose: 2 mg/kg IV, repeat q 5 min until seizure stop (max load 10 mg/kg) Maintenance infusion: 1 to 10 mg/kg/h (< 5 if tx > 48 h)

Administering neuromuscular blocking agents and analgesics facilitates comprehensive general anesthesia after administration of the appropriate anesthetic dose. However, in cesarean sections, the primary consideration is fetal well-being. Given that the fetus has been exposed to the adverse effects of continuous seizures, a strategy involving minimal anesthetic agent use, such as rapid sequence induction, is recommended. This approach minimizes fetal exposure to anesthetic agents while effectively managing maternal seizures; this underscores the need for a nuanced and individualized approach in complex clinical scenarios.

The role of pEEG is nuanced in managing anesthesia during cesarean section in patients with RSE; the patient state index range of the patient was maintained between 40 and 50 during surgery, with no significant fluctuations. However, this does not guarantee the absence of seizure activity during the procedure because pEEG primarily assesses the depth of hypnosis.

Attempts have been made to employ pEEG in evaluating seizure activity. Fàbregas et al explored the potential of pEEG in detecting seizure activity^[7]^; the study revealed that pEEG variables were not significantly influenced by frequent focal seizure activity, highlighting the limitations of pEEG in precisely indicating seizure activity. The limitations of pEEG are significantly displayed in electroconvulsive therapy; during ECT, pEEG for monitoring during anesthesia and conventional EEG for discerning seizure activity were applied. However, pEEG cannot assess the start and end of seizure activity, highlighting its limitations in accurately detecting and evaluating seizure states in such scenarios.

This case reflects a missed opportunity to use a portable wireless continuous electroencephalogram monitoring device in the operating theater, a possibility that could have been realized through collaboration with a neurologist. However, the primary focus of anesthesia in such cases should be the rapid induction and safe delivery of the fetus. While the limited role of pEEG suggests the potential for interdisciplinary cooperation with neurologists or attempts to provide monitored maternal seizure activity during surgery, it is crucial that these measures do not delay the progress of anesthesia and the surgical procedure.

The optimal management of cesarean sections in patients with RSE highlights the necessity of an interdisciplinary approach. Neurologists play a pivotal role in monitoring, essential for the real-time assessment and management of the patient’s seizure activity. Continuous neurological evaluation guides anesthetic and therapeutic decisions throughout the procedure. Pediatricians are crucial in addressing fetal concerns related to the fetus, especially given the potential for exposure to hypoglycemia and hypoxia; their expertise is vital for the immediate assessment and treatment of newborns, ensuring prompt intervention for complications arising from intrauterine distress. Collaboration among obstetricians, anesthesiologists, neurologists, and pediatricians is integral to achieving the best outcomes for mothers and children, illustrating the importance of a cohesive multidisciplinary team in managing such complex cases. Table 2 shows the roles of the anesthesiologists, neurologists, pediatricians, and obstetricians.

**Table 2:** Roles of the anesthesiologists, neurologists, pediatricians, and obstetricians.

Anesthesiologist role Determines the most appropriate method of anesthesia and manages its administration. In cases where the patient is under sedation, the anesthesiologist is responsible for deepening sedation to the level required for general anesthesia, Balancing the need for seizure control with the safety of the mother and the fetus.	Neurologist role The neurologist expertise is vital in managing SE, making informed decisions about sedation levels, and providing cEEG monitoring; this is essential for the real-time assessment and management of the patient neurological status, allowing for tailored anesthetic and therapeutic interventions.
Pediatrician role The pediatrician is pivotal in assessing fetal well-being and managing the newborn airway postdelivery. Their expertise is crucial in ensuring the immediate and effective care of the newborn, particularly in situations where the fetus might have been subjected to stress due to maternal seizures or hypoxia.	Obstetrician role The obstetrician leads the surgical team in performing the cesarean section. They are responsible for the safe delivery of the baby, managing complications that may arise during the surgery, and ensuring the mother well-being throughout the procedure.

cEEG: continuous electroencephalogram, SE: status epilepticus.

One limitation of this case report was that we could not specify an appropriate anesthetic drug dose for such cases. Determination of the dose depends on factors including failure of seizures occurring in mothers to respond to antiepileptic drugs, the duration of exposure of mothers to hypoxia, and the severity of fetal stress. Furthermore, given the lack of evidence to clearly establish a causal relationship, we can only hypothesize that the cognitive impairment was caused by prolonged seizures.

In summary, this case illustrates the challenges faced by anesthesiologists in managing cesarean section in patients with RSE. We recommend that anesthesiologists provide perioperative care by utilizing available hospital resources. The cooperation of a multidisciplinary team is crucial to achieving optimal seizure monitoring and ensuring the safety of mothers and neonates. This study guides the anesthesiology team to deliver proper anesthesia care in complex and demanding scenarios.

## 4. Patient’s guardian’s perspective

Due to the patient’s lack of consciousness and clarity during critical moments, direct insights from the patient are limited. Therefore, it is important to focus on the emotions and decisions of the patient’s guardian. In such emergency scenarios, the process of medical decision-making involves not only the healthcare professionals but also the guardians. Providing them with comprehensive information and convincing them of the appropriateness of the chosen medical interventions is equally crucial.

## Acknowledgments

We would like to thank Editage (www.editage.co.kr) for English language editing.

## Author contributions

**Conceptualization:** Jin Hun Chung.

**Data curation:** Yu Jun Park.

**Formal analysis:** Jun Yong Jung.

**Investigation:** Yong Han Seo.

**Methodology:** Ho Soon Jung.

**Project administration:** Hyung Youn Gong.

**Resources:** Jae Yong Ji.

**Software:** Yu Jun Park.

**Supervision:** Nan Seol Kim.

**Validation:** Jae Yong Ji.

**Visualization:** Jun Yong Jung.

**Writing – original draft:** Jin Soo Park.

**Writing – review & editing:** Hea Rim Chun.
